# Familial hypercholesterolemia in St.-Petersburg: the known and novel mutations found in the low density lipoprotein receptor gene in Russia

**DOI:** 10.1186/1471-2350-6-6

**Published:** 2005-02-08

**Authors:** Faina M Zakharova, Dorte Damgaard, Michail Y Mandelshtam, Valery I Golubkov, Peter H Nissen, Gitte G Nilsen, Anette Stenderup, Boris M Lipovetsky, Vladimir O Konstantinov, Alexander D Denisenko, Vadim B Vasilyev, Ole Faergeman

**Affiliations:** 1 Department of Molecular Genetics, Institute of Experimental Medicine, St.-Petersburg, Russia; 2 Department of Medicine and Cardiology, Aarhus Sygehus, Aarhus University Hospital, Aarhus, Denmark; 3 Department of Clinical Biochemistry, Aarhus Sygehus, Aarhus University Hospital, Aarhus, Denmark; 4 Institute of Human Brain, St.-Petersburg, Russia; 5 Department of Biochemistry, Institute of Experimental Medicine, St.-Petersburg, Russia

## Abstract

**Background:**

Familial hypercholesterolemia is a human monogenic disease caused by population-specific mutations in the low density lipoprotein (LDL) receptor gene. Despite thirteen different mutations of the LDL receptor gene were reported from Russia prior to 2003, the whole spectrum of disease-causing gene alterations in this country is poorly known and requires further investigation provided by the current study.

**Methods:**

Forty-five patients with clinical diagnosis of FH were tested for the apolipoprotein B (apoB) mutation R3500Q by restriction fragment length analysis. After exclusion of R3500Q mutation high-sensitive fluorescent single-strand conformation polymorphism (SSCP) analysis and automatic DNA sequencing were used to search for mutations in the LDL receptor gene.

**Results:**

We found twenty one rare sequence variations of the LDL receptor gene. Nineteen were probably pathogenic mutations, and two (P518P, T705I) were considered as neutral ones. Among the mutations likely to be pathogenic, eight were novel (c.670-671insG, C249X, c.936-940del5, c.1291-1331del41, W422X, c.1855-1856insA, D601N, C646S), and eleven (Q12X, IVS3+1G>A, c.651-653del3, E207X, c.925-931del7, C308Y, L380H, c.1302delG, IVS9+1G>A, V776M, V806I) have already been described in other populations. None of the patients had the R3500Q mutation in the apoB gene.

**Conclusions:**

Nineteen pathogenic mutations in the LDL receptor gene in 23 probands were identified. Two mutations c.925-931del7 and L380H are shared by St.-Petersburg population with neighbouring Finland and several other mutations with Norway, Sweden or Denmark, i.e. countries from the Baltic Sea region. Only four mutations (c.313+1G>A, c.651-653del3, C308Y and W422X) were recurrent as all those were found in two unrelated families. By this study the number of known mutations in the LDL receptor gene in St.-Petersburg area was increased nearly threefold. Analysis of all 34 low density lipoprotein receptor gene mutations found in St.-Petersburg argues against strong founder effect in Russian familial hypercholesterolemia.

## Background

Familial hypercholesterolemia (FH) (OMIM #143890) is one of the most common monogenic human diseases. It is inherited as an autosomal dominant trait with the prevalence of the heterozygous form conventionally considered to be about 1 of 500 in most populations [1]. Elevated blood serum cholesterol is due to impaired removal of low-density lipoproteins (LDL) from blood by LDL receptors, and it is associated with early onset coronary artery disease and myocardial infarction. Impairment of LDL receptor function usually results either from the absence or deficiency of the LDL receptor (OMIM *606945) itself or from a common mutation (R3500Q) in the gene of the receptor's ligand, apolipoprotein B (OMIM +107730) causing type B of the autosomal dominant hypercholesterolemia (OMIM #144010) [2]. Furthermore, a third type of monogenic autosomal dominant hypercholesterolemia (OMIM #603776) is due to the recently discovered defects in the proprotein convertase PCSK9 gene (OMIM *607786) [3]. A form of recessively inherited hypercholesterolemia (OMIM #603813) has a prevalence of less than 1:10 000000 and is due to defects in the LDL receptor adaptive protein ARH (OMIM *605747) [4,5]. Even though many forms of monogenic hypercholesterolemia are known, only apoB gene and LDL receptor gene variations seem to contribute significantly to the CHD morbidity in most populations [6].

The spectrum of LDL receptor mutations varies between different human populations and more than 900 mutations in the LDL receptor gene have been characterized worldwide [7-9]. A recent review [9] shows a clear difference in the LDL receptor gene mutations spectra for Western European countries, but this review gives nearly no data on the genetics of FH in the Eastern European countries and Russia. However, many mutations were described from Poland [10] and Bulgaria [11] and already thirteen different LDL receptor gene mutations have been published from the Russian population prior to 2003 [12]. In the present research we expand the study of the molecular genetic basis for FH in St.-Petersburg known to be the most well studied region of Russia in respect to FH-causing mutations and report 21 mutations, previously unknown in Russia.

## Methods

### Patients

Patients with FH were recruited from two lipid clinics of St.-Petersburg, namely Institute of Human Brain and Institute of Experimental Medicine. The clinical diagnosis of familial hypercholesterolemia was based on the following criteria: highly elevated plasma total cholesterol and LDL-cholesterol, presence of tendon xanthomata, corneal arcus or both, and positive family history of myocardial infarction and hypercholesterolemia with at least one first-degree relative affected. Forty-five probands fulfilling at least two of three criteria listed above were selected for the study. Full data of patients including their lipid data are given in the Discussion section (see Table 2). Informed consent was obtained in each case for DNA testing procedures.

### Biochemical procedures

Genomic DNA was extracted from blood white cells using a standard method [13]. The patients were initially tested for the apoB mutation R3500Q by restriction fragment length analysis [14]. Exons of the LDL receptor gene were then amplified [15], and PCR products were subjected to gel-electrophoresis followed by ethidium bromide or silver staining of DNA to exclude non-specific amplification. Single-strand conformation polymorphism (SSCP) analysis was performed in an ABI 377 DNA Sequencer (PE-Applied Biosystems) sequencing device using 4,25% non-denaturing MDE polyacrylamide gels (Cambrex) at 20°C. The gels were run under two different conditions, i.e. standard MDE gel or with the addition of 5% glycerol to the MDE gel. Automated DNA sequencing was performed in part in the ABI 377 DNA Sequencer (PE-Applied Biosystems), in part in an ALFExpress-2 DNA sequencer (Amersham Life Sciences), using primers for routine PCR DNA amplification. In the case of samples bearing the deletions, PCR products were cloned into a commercially available vector (TACLONE, Medigen, Novosibirsk) and sequenced using universal and reverse primers.

## Results

**Figure 1:**
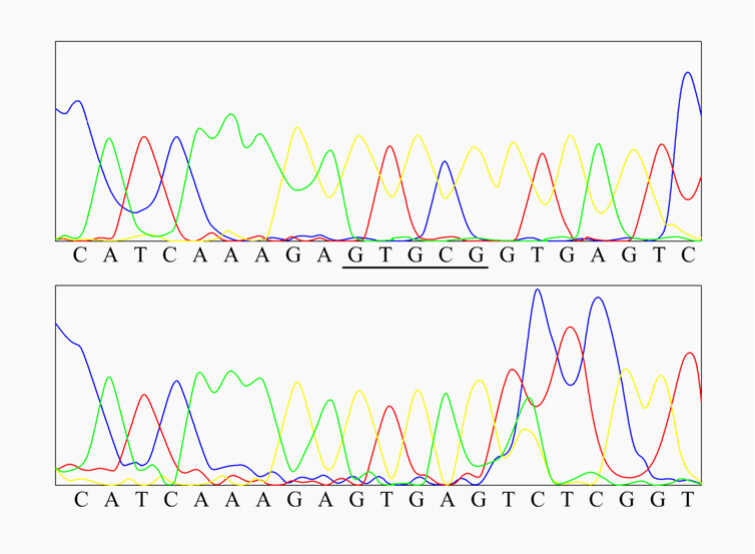
**Sequencing of the cloned mutant allele bearing the five nucleotide deletion (c.936-940del5 or FsE291)**. The sequence of the normal allele is shown at the top. The nucleotides absent in the mutant allele are underlined. The sequence of the mutant allele is shown at the bottom.

None of the 45 patients had the apoB R3500Q mutation, whereas 25 patients had mutations in the LDL receptor gene. Large genomic rearrangements in the LDL receptor gene were previously shown to be an uncommon cause of FH in St.-Petersburg [16] and had been excluded in most patients of the current group. Therefore we restricted our search to point mutations and to minor deletions and insertions. We identified 21 sequence variations (Table 1) of which two probably were not pathogenic (in the following considerations we give numbering of aminoacids in the LDL receptor according to Yamamoto's nomenclature, [17] ). The T705I mutation (known also as FH Paris-9) is not associated with elevated serum cholesterol [18], and the transition c.1617C>T (P518P) is synonymous. We consider the remaining 19 variations to be pathogenic. Eleven mutations have been described in other populations, but to our knowledge the remaining 8 mutations have not been described so far [7,8]. From two of these nucleotide substitutions (C249X and W422X) the stop codon arises, four are frame-shift mutations leading to premature stop codons (FsK202:S205X; FsK290:N309X; FsV409:S423X; FsV597:A622X), and two result in amino acid substitutions (D601N and C646S). Sequencing of the cloned mutant allele bearing the five-nucleotide deletion (c.936-940del5 or FsE291) is demonstrated on Fig. 1. Mutations c.313+1G>A, c.651-653del3, C308Y and W422X were found in two probands each.

**Figure 2:**
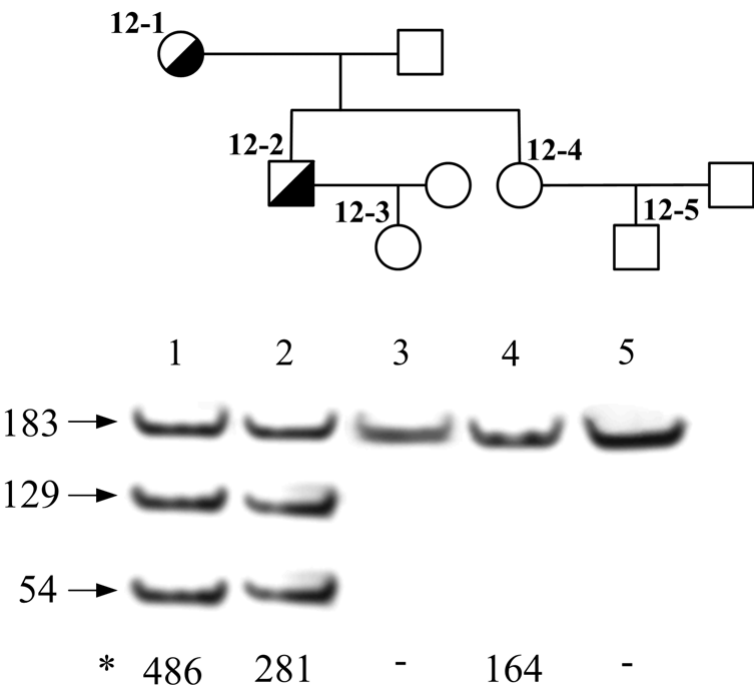
**Detection of mutation Q12X in the LDL receptor gene by means of *Mae I *restriction enzyme test**. Transition c.97 C>T in exon 2 of the LDL receptor gene leads to appearance of the new *Mae I *restriction site (CTAG) in the patients with the mutation. *Mae I *restriction enzyme test enables to confirm the presence of mutation Q12X identified by DNA sequencing in the proband (12-1) and in her son (12-2) and to exclude presence of the mutation in other relatives of the proband, including daughter (12-4) and grandchildren (12-3 and 12-5). Lengths of DNA restriction fragment are given at the left in bp and *total blood serum cholesterol figures of the patients – at the bottom of the gel in mg/dl.

**Figure 3:**
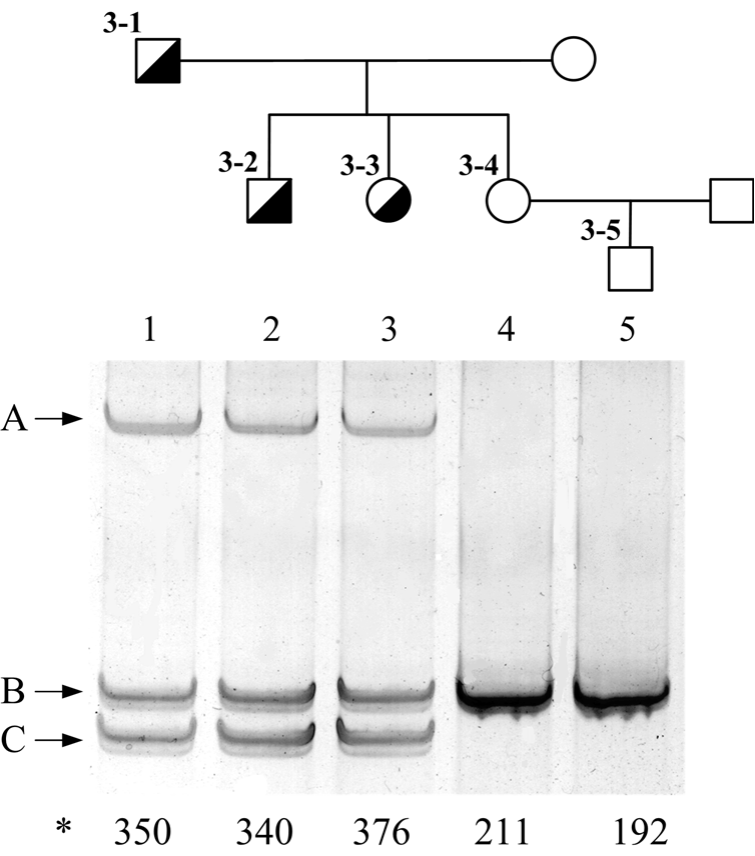
**Detection of mutation c.1291-1331del41 (mutation FsV409:S423X) of the LDL receptor gene by means of PCR product sizing and heteroduplex analysis**. Deletion of 41 nucleotides results in significant change (indicated by C) of molecular weight of the PCR-amplified LDL receptor gene exon 9 fragment and in formation of specific heteroduplexes (indicated by A). Letter B indicates the PCR product of normal size. Current gel supports the presence of c.1291-1331del41 mutation in two children (3-2, 3-3) of the proband (3-1) and absence of this mutation in his daughter and grandson (3–4, 3–5). *total blood serum cholesterol figures of the patients – at the bottom of the gel in mg/dl.

Rapid tests were developed for most of the mutations and all of those were confirmed by using these methods (Table 1). Various detection methods, including heteroduplex analysis, restriction enzyme tests and SSCP for several mutations from the list are illustrated by Figures 2, 3. Cosegregation of the mutations and elevated blood serum cholesterol was demonstrated in seventeen families (see Fig. 2, 3 and Table 1). Totally the mutations were confirmed in 9 relatives and excluded in 27 members of the proband's families. Most important the diagnosis of FH was excluded in 9 and set in 2 children of probands before adulthood, i.e. prior to age 18. In these children mutation detection was of crucial importance to set the diagnosis and to suggest further life style.

In case of the Q12X mutation, the c.97C>T transition leads to occurrence of a new Mae I restriction site (CTAG) in exon 2 that allows a simple detection of this mutation. Restriction enzyme test was performed for the proband and her 4 descendants (Fig. 2). The presence of the mutation was confirmed in the son of the proband and excluded in the daughter and two grandchildren.

In some other mutations no restriction enzyme tests can be developed for their rapid detection. For example, it is true for the recurrent mutation IVS3+1G>A (c.313+1G>A) that was identified by means of SSCP and verified by sequencing in probands from two unrelated families. SSCP patterns on silver-stained gels differ strikingly in patients with and without the mutation. The presence of the mutation was confirmed in the son of the proband by SSCP.

In heterozygotes a deletion of 41 nucleotides at c.1291-1331 in exon 9 (mutation FsV409:S423X) results in occurrence of specific heteroduplexes. Besides, a PCR fragment of smaller size as compared to the normal allele is revealed in silver-stained polyacrylamide gels (Fig. 3). This mutation was identified in the son and daughter of the proband and excluded in the second daughter and in her child (Fig. 3).

## Discussion

Previously 13 mutations in the LDL receptor gene have been reported in FH patients residing in St.-Petersburg [12] (see Table 3). Eight of those have not been reported in other populations. Our study revealed 21 mutations in the LDL receptor gene, out of which only T705I was previously reported from St.-Petersburg [12]. Nineteen of those 21 mutations are likely to be pathogenic. We exclude P518P (CCC>CCT; c.1617 C>T) from the list of pathogenic mutations since the transition c.1617 C>T results neither in an amino acid substitution nor in appearance of the new consensus splicing sequences. P518P mutation was found in the proband with mutation C646S, but it was not clarified if the mutations were in cis- or trans- position. Also we consider the T705I variant not to be a primary cause of FH, since the I705 allele itself is not associated with elevated cholesterol level [18] and the probable hypercholesterolemic effect of this mutation may be due to its linkage with other pathogenic LDL receptor gene mutations. Indeed, a variation in intron 7 (c.1061-8T) of unclear functional significance was shown to be very tightly linked to the I705 allele [19,20]. We have not searched for this intronic variant in the LDL receptor in the patient with the T705I substitution.

Among pathogenic mutations reported here, eleven have been described in other populations (Table 1) [7,8], and eight are novel. Six of the novel mutations lead to premature stop codons and result in truncated protein chains that probably lose their function. One out of these truncating mutations (c.936-940del5 or FsE291) also changes invariant nucleotides nearby exon-intron junction and thus may affect splicing. The D601N missense mutation, causing substitution of aspartic acid by asparagine has not been reported before. It seems likely that such a substitution might cause the loss of function since one other mutation in the same codon (D601Y) was described in familial hypercholesterolemia subjects [7]. The C646S also has not been reported before, but 5 other mutations affecting this codon have been described, one of which, C646Y (FH French Canadian-2), results in a transport defective protein (mutation class 2A) [21]. We, therefore, find it very likely that the C646S mutation is also pathogenic, but expression studies are needed to justify the effect of both missense mutations D601N and C646S. Previously described mutations were considered to be pathogenic due mostly to their listing in FH mutation databases even though functional studies were not systematically performed. Indeed, V806I mutation (known as variant FH New York -5) [22] occurs in the LDL receptor internalization signal NPVY, for which the consensus sequence NPxY is given (where X is not a conserved aminoacid) [23] and thus the substitution of isoleucin for valine may be not crucial for the LDL receptor function. The V776M mutation may have effect on LDL receptor mRNA splicing rather than to be realized on the protein level since the V776M mutation changes the invariant G at the 3' end of exon 16. However, this mutation is likely to be pathogenic since it was reported already in patients from La Habana, Cuba [24].

The apoB R3500Q mutation was not detected in any of our patients. This finding is in agreement with the previous observation that the R3500Q mutation had not been found in St.-Petersburg [25]. The mutation has been detected in another part of Russia only in 2 of 71 patients with symptoms of familial hypercholesterolemia [26]. The apoB R3500Q mutation is almost exclusively found in Caucasian individuals, and almost all subjects with the mutation carry the same haplotype. One of the highest frequencies of the mutation has been found in the Swiss population (approximately 1/200) [27], and Miserez and Muller [27] hypothesized that the mutation may have arisen in Switzerland 10,000 – 6,000 years ago. The prevalence of the mutation declines with increasing distance from the Central Europe [27,28], and the prevalence of the apoB R3500Q mutation is therefore expected to be low in the St.-Petersburg area (< 1/1000). A precise estimate would require a random sampling of the general population.

Mutations in LDL receptor gene typical for various ethnic groups were revealed in St.-Petersburg (Table 1). In particular, several mutations found in Denmark, Finland, Norway and Sweden are present in the St.-Petersburg population. The ethnic origin of these families is unclear and according to questioning and family names no evidence of a Scandinavian origin of probands was obtained. Only exception from our previous FH group was the proband with the E207X mutation who stated his German roots and indeed this mutation was previously reported from several families from Germany [29]. Interestingly, no specific Slavic or Eastern-European founder mutations were found in St.-Petersburg when comparing the LDL receptor mutation spectrum of Russia to those of Poland [10], Bulgaria [11] or Czech Republic [30,31]. G571E was found in Russia, Czech Republic and Poland but this mutation was also reported from many other countries worldwide. The C188Y mutation found in Russia [32] and in Czech Republic [30,31] cannot be considered a founder Slavic mutation, since it was reported from unique families in each country. Only one candidate for a Russian founder mutation is C139G identified in four unrelated families in different regions of the country, but this mutation is absent in related nations of the Eastern Europe as well as in other countries in the world.

In our study we were able to find LDL receptor gene pathogenic mutations in 23 of 45 patients. Many methods are considered to be superior to routine isotopic SSCP when screening for mutations in DNA (see e.g. [33]for comparison of SSCP and DHPLC). However, we believe that mutations could have been overlooked due to intrinsic limitations of SSCP method in our hands only in few cases. Fluorescent SSCP, used in the current study, can be superior even to DHPLC and is a sensitive method for mutation screening, especially when different gel electrophoresis conditions are applied [34]. Recent studies indicate, that SSCP run under two different conditions detect up to 96% of heterozygous variations (P.H. Nissen, unpublished results). In our study LDL receptor gene mutations were found in 56% (14 out of 26) patients selected by SSCP and in 53% (10 out of 19) patients which DNA was subjected to direct sequencing of all gene exons (Table 2). We do not find it likely that many patients are underdiagnosed due to presence of large genomic rearrangements. In a study conducted in St.-Petersburg, in the partly overlapping sample of FH patients, only one case of a large deletion was found in the sample of 50 probands, giving the rough estimate of genomic rearrangements 2% [16]. In fact in mixed populations (such as that of that of St. Petersburg) the contribution of large rearrangements to the spectrum of pathogenic mutations seems to vary from 6% in Great Britain [35] to 2.5% in English-speaking Canadians [36]. More important, some of intronic mutations leading to defects of splicing could be missed, since the design of primers [15] mostly following recommendations of Hobbs et al. [22] with the only exclusion for primers to amplify exon 3 allowed analyzing only 14 out of 34 intron-exon boundaries in the LDL receptor gene. Recent investigation [37] demonstrated that a high percent of previously missed LDL receptor mutations may be localized in introns. In the cited study [37] in the patient sample after modifying the gene analysis procedure nearly 27% of patients turned out to have intronic mutations, despite the functional significance of these mutations have still to be validated.

In the apoB gene we only looked for the R3500Q mutation, and the possibility that other mutations in that gene are responsible for hyperlipidemia cannot be ruled out. This possibility is unlikely, since the apoB gene has been studied extensively in other laboratories and no other pathogenic variants have been determined. To conclude we cannot definitely rule out the possibility that some undiscovered mutations in the LDL receptor gene have not been found. However, the possibility that the patients have mutations in a different gene involved in FH still remains since the mutation in the PCSK9 gene [3] were not tested in this study.

With the two exceptions, mutations previously reported from Russia [12,38] have been confined to a single family. The exceptions were the mutations G197del (FH-Lithuania), found in high percent of Ashkenazi Jewish families with FH from St.-Petersburg [39], and the C139G mutation found in two Slavic families from St.-Petersburg [40,12], one family in Novosibirsk [38] and one family in Moscow [41]. In this study 4 mutations, namely c.313+1G>A, c.651-653del3, C308Y and W422X were found in two unrelated families. Together with the previous findings we discovered 34 mutations in the LDL receptor gene in St. Petersburg of which only six were detected in more than one family. Fourteen LDL receptor gene mutations were discovered by the other Russian FH team from Moscow, Russia out of which only C139G is shared with St.-Petersburg population [12,41]. To date a total of 47 different FH mutations are known from Russia. The data presented here enlarge the spectrum of mutations found in the Russian population and make us regard it as genetically heterogeneous.

## Conclusions

We identified nineteen pathogenic mutations in the LDL receptor gene in 23 probands and two probably neutral mutations. In our study only four mutations in the LDL receptor gene (c.313+1G>A, c.651-653del3, C308Y and W422X) were found to be recurrent, i.e. all of those were found in two apparently unrelated families. Together with the data obtained earlier [12,38] our results present an evidence against the strong founder effect among Russian FH patients, and it is likely that in the St.-Petersburg area there is as much genetic heterogeneity as in most other areas of the world.

## Competing interests

The author(s) declare that they have no competing interests.

## Authors' contributions

FMZ, MYM, VIG, PHN, GGN, AS performed cloning, SSCP, sequencing and familial analysis, BML, VOK, DD, ADD selected patients, VBV and OF participated in the study design and coordination. All authors read and approved the final manuscript.

## Pre-publication history

The pre-publication history for this paper can be accessed here:



## Figures and Tables

**Table 1:** List of the LDL receptor gene mutations found in the current study

**Mutation, systematic name**	**Nucleotide change**	**Exon/Intron**	**Rapid test method**	**Occurrence in other populations [7, 8]**	**Number of families (patients) with the mutation**
**Missense-mutations**					

C308Y	c.985 G>A	exon 7	*FblI*	China	2 (2)
L380H (FH Pori)	c.1202 T>A	exon 9	*MnlI*	Finland	1 (1)
D601N	c.1864 G>A	exon 13	*EcoRV*	None (New)	1 (2)
C646S	c.1999 T>A	exon 14	SSCP	None (New)	1 (1)
V776M	c.2389 G>A	exon 16	SSCP	China (Hong-Kong), Cuba, South Africa (Afrikaners)	1 (2)
V806I (FH New York-5)	c.2479 G>A	exon 17		The Netherlands, USA	1 (1)

**Nonsense-mutations**					

Q12X (FH Turkey/Milan-4)	c.97 C>T	exon 2	*MaeI*	Italy, France, Turkey	1 (2)
E207X (FH Morocco)	c.682 G>T	exon 4	*MaeI*	China, Germany, Korea, Morocco, Norway, Sweden, UK, USA	1 (2)
C249X	c.810 C>A	exon 5	*DdeI*, *Fnu4HI*	None (New)	1 (1)
W422X	c.1328 G>A	exon 9	*MaeI*	None (New)	2 (3)

**Splice site mutations**					

IVS3+1G>A (FH-Elverum/ Olbia)	c.313+1G>A	intron 3	SSCP	Austria, Belgium, Denmark, Germany, Italy, Spain, Korea, Norway, The Netherlands, UK, Sweden, South Africa (black)	2 (3)
IVS9+1G>A	c.1358+1G>A	intron 9	*AsuHPI*	The Netherlands	1 (1)

**Frameshift mutations**					

FsK202: S205X	c.670-671insG	exon 4	SSCP	None (New)	1 (2)
FsE287: V348X (FH North Karelia)	c.925-931del7	exon 6	SSCP	Finland, Sweden, USA	1 (1)
FsE291: N309X	c.936-940del5	exon 6	SSCP	None (New)	1 (1)
FsV409: S423X	c.1291-1331del41	exon 9	Sizing	None (New)	1 (3)
FsE414: M429X	c.1302delG	exon 9	SSCP	Germany	1 (1)
FsV597: A622X	c.1855-1856insA	exon 13		None (New)	1 (1)

**In-frame deletions**					

G197del	c.651-653del3	exon 4	HA	UK	2 (2)

**Neutral mutations**					

T705I (FH Paris-9)	c.2177 C>T	exon 15		Denmark, France, The Netherlands, UK, USA etc.	1 (1)

**Silent mutations**					

P518P	c.1617 C>T	exon 11		None (New)	1 (1)

Footnote: HA – heteroduplex analysis; SSCP – single-strand conformation polymorphism analysis. Numeration of nucleotides and aminoacids follows Yamamoto's nomenclature [17] and the letter c. before the number of nucleotide indicates that it was taken from cDNA sequence.

**Table 2:** Clinical features of St. Petersburg patients recruited for search of mutations in the LDL receptor gene

**Patients ID**	**Sex**	**Age**	**TC, mg/dl**	**TG, mg/dl**	**LDLC, mg/dl**	**HDLC, mg/dl**	**Xanthomas/Corneal arcus**	**CHD**	**CI**	**Mutation name**	**All exons sequencing**
**1**	F	61	449	178	386	27	+	+	+	V776M	-
**2**	F	58	675	305	572	42	+	+	-	T705I	-
**3**	M	60	350	81	284	50	+	+	-	FsV409: S423X	-
**4**	F	49	326	203	248	37	-	+	-	-	-
**5**	F	36	414	201	321	53	+	-	-	V806I	-
**6**	M	68	404	192	333	33	+	+	-	-	-
**7**	F	76	522	161	444	46	-	-	-	-	-
**8**	F	54	446	165	353	60	-	-	-	-	-
**9**	F	52	500	ND	ND	ND	-	+	+	-	-
**10**	F	62	370	120	303	43	+	-	-	C308Y	-
**11**	F	66	359	141	300	31	-	+	-	-	-
**12**	F	66	486	296	391	36	-	+	+	Q12X	-
**13**	F	52	469	228	389	34	+	+	-	IVS9+1G>A	-
**14**	F	53	325	101	286	19	-	+	+	-	-
**15**	F	58	446	192	334	74	-	+	-	FsK202: S205X	-
**16**	F	38	398	260	314	32	+	-	-	FsE291: N309X	-
**17**	M	46	280	117	220	37	-	+	+	-	-
**18**	F	58	519	179	446	37	-	+	-	G197del	-
**19**	F	54	364	135	300	37	+	+	+	FsE287: V348X	-
**20**	F	53	204	233	135	23	-	+	+++	-	-
**21**	M	61	350	116	280	47	-	-	-	-	-
**22**	F	48	369	221	290	35	-	+	-	-	-
**23**	M	48	417	190	353	30	-	+	-	C249X	-
**24**	F	45	299	122	229	46	+	-	-	IVS3+1G>A	-
**25**	F	50	353	101	295	38	+	-	-	FsE414: M429X	-
**26**	F	45	558	210	478	38	+	+	+	-	-
**27**	M	62	469	136	398	44	+	+	++	G197del	+
**28**	F	8	915	179	850	29	+	-	-	-	+
**29**	F	40	367	73	283	69	-	+	-	W422X	+
**30**	F	60	480	104	393	66	-	-	-	-	+
**31**	M	41	314	247	226	39	-	+	+	-	+
**32**	M	37	344	292	262	24	+	+	+	-	+
**33**	F	52	360	744	179	32	+	+	+	-	+
**34**	M	40	312	70	258	40	+	+	++	-	+
**35**	M	29	253	79	195	42	-	-	-	E207X	+
**36**	M	38	520	168	447	39	+	+	+	D601N	+
**37**	F	19	447	156	362	54	-	-	-	L380H	+
**38**	F	63	347	84	266	64	+	+	+	C646S	+
**39**	F	56	432	106	374	37	+	+	-	W422X	+
**40**	M	47	540	226	446	49	+	+	+	IVS3+1G>A	+
**41**	F	70	433	123	370	38	-	+	+	-	+
**42**	M	34	534	ND	ND	ND	+	+	+	-	+
**43**	F	52	654	115	573	58	+	+	+	C308Y	+
**44**	M	52	356	334	260	29	-	+	+	-	+
**45**	M	52	360	169	287	39	+	+	+	FsV597: A622X	+

Footnote: TC – total blood serum cholesterol, TG – triglycerides, LDLC – low density lipoprotein cholesterol, HDLC – high density lipoprotein cholesterol, CHD – coronary heart disease, CI – coronary infarction. Pluses in the CI column indicate number of CI survived. Minus in the right column indicates that the patient's LDL receptor gene was studied by high-sensitive fluorescent SSCP-analysis and not by frontal sequencing of all LDL receptor gene exons; the latter condition is indicated by plus.

**Table 3:** List of the LDL receptor gene mutations reported previously from St. Petersburg, Russia [12 and References therein]

**Mutation, systematic name**	**Nucleotide change**	**Exon/Intron**	**Rapid test method**	**Occurrence in other populations [7, 8]**	**Number of families (patients) with the mutation**
**Large rearrangements**					

delta 5kb	Large deletion	exons 3-5	Southern blot	None	1 (2)

**Missense-mutations**					

C127W	c.444 T>G	exon 4	*Mva I*	None	1 (1)
G128G, A130P	[c.447 T>C; c.451 G>C]	exon 4	*Cac 8 I, Bsu R I*	None	1 (1)
C139G	c.478 T>G	exon 4	*Msp I*	None	1 (3)
C146R	c.499 T>C	exon 4	*Apa I*	None	1 (3)
C188Y	c.626 G>A	exon 4	*Rsa I*	Czech Republic	1 (3)
G571E	c.1775 G>A	exon 12	SSCP	Italy, Germany, Poland, Czech Republic, Austria, Belgium, Greece	1 (1)

**Nonsense-mutations**					

C74X	c.285 C>A	exon 3	*DdeI*	Korea, Northern Japan	1 (1)
E397X	c.1252 C>T	exon 9	*Alu I*	None	1 (7)

**In-frame deletions**					

347delGCC	c.347-349del	exon 4	HA, *Fnu4HI*	None	1 (1)
G197del	c.652-654del	exon 4	HA	Israel, USA, UK, Poland, Czech Republic, Germany, South Africa, The Netherlands	7 (14)

**Neutral mutations**					

T705I (FH Paris-9)	c.2177 C>T	exon 15		Denmark, France, The Netherlands, UK, USA etc.	1 (1)

**Silent mutations**					

H229H	c.750 C>T	exon 5	*Nco I*	None (New)	1 (1)

Footnote: HA – heteroduplex analysis; SSCP – single-strand conformation polymorphism analysis. Numeration of nucleotides and aminoacids follows Yamamoto's nomenclature [17] and the letter c. before the number of nucleotide indicates that it was taken from cDNA sequence.

## References

[B1] Goldstein JL, Hobbs HH, Brown MS, Scriver CR, Beaudet AL, Sly WS, Vale D (2001). Familial hypercholesterolaemia. The metabolic and molecular basis of inherited disease.

[B2] Innerarity TL, Mahley RW, Weisgraber KH, Bersot TP, Krauss RM, Vega GL, Grundy SM, Friedl W, Davignon J, McCarthy BJ (1990). Familial defective apolipoprotein B-100: a mutation of apolipoprotein B that causes hypercholesterolemia. J Lipid Res.

[B3] Abifadel M, Varret M, Rabes JP, Allard D, Ouguerram K, Devillers M, Cruaud C, Benjannet S, Wickham L, Erlich D, Derre A, Villeger L, Farnier M, Beucler I, Bruckert E, Chambaz J, Chanu B, Lecerf JM, Luc G, Moulin P, Weissenbach J, Prat A, Krempf M, Junien C, Seidah NG, Boileau C (2003). Mutations in PCSK9 cause autosomal dominant hypercholesterolemia. Nat Genet.

[B4] Garcia CK, Wilund K, Arca M, Zuliani G, Fellin R, Maioli M, Calandra S, Bertolini S, Cossu F, Grishin N, Barnes R, Cohen JC, Hobbs HH (2001). Autosomal recessive hypercholesterolemia caused by mutations in a putative LDL receptor adaptor protein. Science.

[B5] Soutar AK, Naoumova RP, Traub LM (2003). Genetics, clinical phenotype, and molecular cell biology of autosomal recessive hypercholesterolemia. Arterioscler Thromb Vasc Biol.

[B6] Goldstein JL, Brown MS (2001). The cholesterol quartet. Science.

[B7] The low density lipoprotein receptor (LDLR) gene in familial hypercholesterolemia. http://www.ucl.ac.uk/fh/.

[B8] Universal Mutation Database. http://www.umd.necker.fr/LDLR/Home_Page.html.

[B9] Dedoussis GVZ, Schmidt H, Genschel J (2004). LDL-receptor mutations in Europe. Hum Mutat.

[B10] Gorski B, Kubalska J, Naruszewicz M, Lubinski J (1998). LDL-R and Apo-B-100 gene mutations in Polish familial hypercholesterolemias. Hum Genet.

[B11] Mihaylov VA, Horvath AD, Savov AS, Kurshelova EF, Paskaleva ID, Goudev AR, Stoilov IR, Ganev VS (2004). Screening for point mutations in the LDL receptor gene in Bulgarian patients with severe hypercholesterolemia. J Hum Genet.

[B12] Low density lipoprotein receptor gene mutations in Russian patients with familial hypercholesterolemia. http://www.iemrams.spb.ru/english/molgen/fh-en/fh-rusen.htm.

[B13] Kunkel LM, Smith KD, Boyer SH, Borgaonkar DS, Wachtel SS, Miller OJ, Breg WR, Jones HW, Rary JM (1977). Analysis of human Y-chromosome-specific reiterated DNA in chromosome variants. Proc Natl Acad Sci U S A.

[B14] Hansen PS, Rudiger N, Tybjaerg-Hansen A, Faergeman O, Gregersen N (1991). Detection of the apoB-3500 mutation (glutamine for arginine) by gene amplification and cleavage with MspI. J Lipid Res.

[B15] Jensen HK, Jensen LG, Hansen PS, Faergeman O, Gregersen N (1996). High sensitivity of the single-strand conformation polymorphism method for detecting sequence variations in the low-density lipoprotein receptor gene validated by DNA sequencing. Clin Chem.

[B16] Mandelshtam MJu, Lipovetskyi BM, Schwartzman AL, Gaitskhoki VS (1993). A novel deletion in the low density lipoprotein receptor gene in a patient with familial hypercholesterolemia from Petersburg. Hum Mutat.

[B17] Yamamoto T, Davis CG, Brown MS, Schneider WJ, Casey ML, Goldstein JL, Russell DW (1984). The human LDL receptor: A cysteine-rich protein with multiple Alu sequences in its mRNA. Cell.

[B18] Lombardi P, Sijbrands EJ, Kamerling S, Leuven JA, Havekes LM (1997). The T705I mutation of the low density lipoprotein receptor gene (FH Paris-9) does not cause familial hypercholesterolemia. Hum Genet.

[B19] Heath KE, Whittall RS, Miller GJ, Humphries SE (2000). I705 variant in the low density lipoprotein receptor gene has no effect on plasma cholesterol levels. J Med Genet.

[B20] Mozas P, Cenarro A, Civeira F, Castillo S, Ros E, Pocovi M (2000). Mutation analysis in 36 unrelated Spanish subjects with familial hypercholesterolemia: identification of 3 novel mutations in the LDL receptor gene. Hum Mutat.

[B21] Leitersdorf E, Tobin EJ, Davignon J, Hobbs HH (1990). Common low-density lipoprotein receptor mutations in the French Canadian population. J Clin Invest.

[B22] Hobbs HH, Brown MS, Goldstein JL (1992). Molecular genetics of the LDL receptor gene in familial hypercholesterolemia. Hum Mutat.

[B23] Chen WJ, Goldstein JL, Brown MS (1990). NPXY, a sequence often found in cytoplasmic tails, is required for coated pit-mediated internalization of the low density lipoprotein receptor. J Biol Chem.

[B24] Pereira E, Ferreira R, Hermelin B, Thomas G, Bernard C, Bertrand V, Nassiff H, Mendez del Castillo D, Bereziat G, Benlian P (1995). Recurrent and novel LDL receptor gene mutations causing heterozygous familial hypercholesterolemia in La Habana. Hum Genet.

[B25] Shevtsov SP (1996). The APOB gene encoding putative low density lipoprotein receptor binding domain of the ApoB-100 protein shows no DNA polymorphisms. Genetika.

[B26] Krapivner SR, Malyshev PP, Rozhkova TA, Poltaraus AB, Kukharchuk VV, Bochkov VN (2000). [Application of DNA analysis for differential diagnosis of familial hypercholesterolemia and familial defect of apolipoprotein B-100]. Ter Arkh.

[B27] Miserez AR, Muller PY (2000). Familial defective apolipoprotein B-100: a mutation emerged in the mesolithic ancestors of Celtic peoples?. Atherosclerosis.

[B28] Horvath A, Ganev V (2001). The mutation APOB-100 R3500Q in Eastern Europe. Atherosclerosis.

[B29] Nauck MS, Koster W, Dorfer K, Eckes J, Scharnagl H, Gierens H, Nissen H, Nauck MA, Wieland H, Marz W (2001). Identification of recurrent and novel mutations in the LDL receptor gene in German patients with familial hypercholesterolemia. Hum Mutat.

[B30] Kuhrova V, Francova H, Zapletalova P, Freiberger T, Fajkusova L, Hrabincova E, Slovakova R, Kozak L (2001). Spectrum of low density lipoprotein receptor mutations in Czech hypercholesterolemic patients. Hum Mutat.

[B31] Kuhrova V, Francova H, Zapletalova P, Freiberger T, Fajkusova L, Hrabincova E, Kozak L, Slovakova R (2002). Spectrum of low density lipoprotein receptor mutations in Czech hypercholesterolemic patients. Hum Mutat.

[B32] Tatishcheva YuA, Mandelshtam MYu, Golubkov VI, Lipovetsky BM, Gaitskhoki VS (2001). Four new mutations and two polymorphic variants of the low-density lipoprotein receptor gene in familial hypercholesterolemia patients from St. Petersburg. Rus JGenet.

[B33] Bunn CF, Lintott CJ, Scott RS, George PM (2002). Comparison of SSCP and DHPLC for the detection of LDLR mutations in a New Zealand cohort. Hum Mutat.

[B34] Ellis LA, Taylor CF, Taylor GR (2000). A comparison of fluorescent SSCP and denaturing HPLC for high throughput mutation scanning. Hum Mutat.

[B35] Horsthemke B, Dunning A, Humphries S (1987). Identification of deletions in the human low density lipoprotein receptor gene. J Med Genet.

[B36] Langlois S, Kastelein JJP, Hayden MR (1988). Characterization of six partial deletions in the low-density-lipoprotein (LDL) receptor gene causing familial hypercholesterolemia (FH). Am J Hum Genet.

[B37] Amsellem S, Briffaut D, Carrie A, Rabes JP, Girardet JP, Fredenrich A, Moulin P, Krempf M, Reznik Y, Vialettes B, de Gennes JL, Brukert E, Benlian P (2002). Intronic mutations outside of Alu-repeat-rich domains of the LDL receptor gene are a cause of familial hypercholesterolemia. Hum Genet.

[B38] Mandelshtam MYu (2003). What were the outcomes of familial hypercholesterolemia studies for understanding of the hyperlipidemia genetics?. Meditsinskaya Genetika.

[B39] Mandelshtam M, Chakir Kh, Shevtsov S, Golubkov V, Skobeleva N, Lipovetsky B, Konstantinov V, Denisenko A, Gaitskhoki V, Schwartz E (1998). Prevalence of Lithuanian mutation among St.-Petersburg Jews with familial hypercholesterolemia. Hum Mutat.

[B40] Chakir Kh, Skobeleva NA, Shevtsov SP, Konstantinov VO, Denisenko AD, Schwartz EI (1998). Two novel Slavic point mutations in the low-density lipoprotein receptor gene in patients with familial hypercholesterolemia from St. Petersburg, Russia. Mol Genet Metab.

[B41] Meshkov AN, Stambol'skii DV, Krapivner SR, Bochkov VN, Kukharchuk VV, Malyshev PP (2004). [Low density lipoprotein receptor gene mutations in patients with clinical diagnosis of familial hypercholesterolemia]. Kardiologiia.

